# Timing of ovulation in the fat‐tailed Qezel ewes after synchronization with vaginal devices containing endogenous or exogenous synthetic progestogens during out of the breeding season

**DOI:** 10.1002/vms3.1269

**Published:** 2023-09-19

**Authors:** Alireza Hamisi, Mohsen Eslami, Farhad Farrokhi‐Ardabili, Sina Bahmani

**Affiliations:** ^1^ Department of Theriogenology Faculty of Veterinary Medicine Urmia University Urmia Iran; ^2^ Department of Animal Sciences Faculty of Agriculture Urmia University Urmia Iran

**Keywords:** CIDR, laparoscopic insemination, oestrus synchronization, ovulation time, vaginal sponge

## Abstract

**Background:**

Wide range of ovulation distribution is the main restricting factor in establishing the pregnancy following oestrus synchronization (ES) and fixed time insemination (FTI) in sheep.

**Objectives:**

Determining the ovulation time (OVT) following ES with two different vaginal devices, its relation to progesterone and conception upon FTI with frozen/thawed semen.

**Methods:**

Oestrus was synchronized using either controlled internal drug release (CIDR) (ewe, *n* = 6; ewe lamb, *n* = 5) or vaginal sponge (ewe, *n* = 6; ewe lamb, *n* = 5) insertion for 12 days, plus Equine chorionic gonadotropin (eCG) at devices removal (Day 0). Sizes of the ovarian follicles were measured using transvaginal probe at −12, 0 and 30–33, 53 h and continued every 3–4 h until 75 h after eCG treatment. Serum progesterone amounts were measured at −12, 0, +2 and +11. Laparoscopic FTI was done at 60.5 ± 0.5 h.

**Results:**

The CIDR‐treated group initiated and completed ovulations earlier compared to sponge‐treated females (median: 64 vs. 71 h; *p* < 0.05). Ewe lambs were ovulated earlier compared to ewes in the sponge‐treated group (66.71 vs. 71.5; *p* = 0.017). Mean sizes of ovulatory follicles and corpora lutea were not affected by device types. Higher amounts of progesterone were observed in CIDR group compared to sponge‐treated group at device removal (2.68 ± 0.12 vs. 0.30 ± 0.01 ng/mL; *p* < 0.001). The conception was confirmed in 2/10, and 5/11 females of sponge and CIDR‐treated females, respectively.

**Conclusions:**

Types of progestogens influence the OVT, and consequently the result of FTI with frozen/thawed semen. The optimum timespan for FTI should be chosen according to the device types during non‐breeding season.

## Graphical abstract

- Numbers and sizes of ovulatory follicles were not affected by the intravaginal device types.
- Ovulation was happened earlier in CIDR‐treated compared to sponge received females.
- Fixed time intrauterine insemination with frozen‐thawed samples near the time of ovulation resulted in greater conception in fat‐tailed females out of the breeding season.

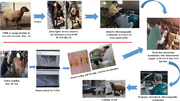

## 1 INTRODUCTION

Given the increased demand for sheep products, including wool, meat and milk in the current century, efforts are focused on the adopting novel and innovative methods for providing the protein needs of the population. Adequate production of animalʼs meat cannot be realized except through the correct livestock breeding (Dias et al., 2020). In this regard, an artificial insemination (A.I.) is an effective tool for the success and improvement of the livestock industry (Salamon & Maxwell, 1995). The use of frozen semen samples of rams provides permanent access to spermatozoa, the possibility of transferring better genetics between countries, and avoiding sexually transmitted diseases. Therefore, the use of frozen spermatozoa of elite rams for A.I. has increased in demand nowadays (Sathe, 2018). However, successful performance of A.I. in the sheep depends on the synchronization of ovulation, and the insemination techniques (Reyna et al., 2007). Hence, fixed timed artificial insemination (FTAI) being carried out following hormone therapy, which minimizes the differences in the ovulation distribution between females and increases the fertility rate (Evans & Maxwell, 1987). Oestrus synchronization (ES) is usually performed through intravaginal administration of progestagen‐containing devices for 7–14 days (Menchaca & Rubianes, 2004). Progesterone inhibits the release of LH from the anterior pituitary gland, resulting in the preventing the final development of ovarian antral follicles and ovulation (Oliveira et al., 2016). Equine chorionic gonadotropin (eCG) is usually used during and beyond the breeding season to stimulate follicular growth, and induction of ovulation in ewes (Silveira et al., 2021).

The controlled internal drug release (CIDR) and vaginal sponges are the two usual intravaginal devices containing progestagen which utilizes in the breeding programs for females (Ainsworth & Shrestha, 1983; Hamra et al., 1989; Swelum et al., 2018). Utilization of vaginal devices plus eCG has usually rendered an acceptable rate of pregnancy following natural breeding. However, diverse ovulation time (OVT) is the main restricting factor in the formation of zygotes and establishing the pregnancy following FTAI, especially with frozen/thawed semen samples. The diversity in OVT may arise from external (treatment with eCG, the mode of FSH preparation or the drug doses, status of nutrition, body condition score, age of ewe and the presence of ram) or the internal factors (ovarian status, genetic diversity and the breed of animal; Walker et al., 1989; Ginther & kot, 1994; Gibbons et al., 1999; Viñoles et al., 1999; Salehi et al., 2010). However, even when these factors are minimized in the treated animals, the OVT is still very changeable in ewes and ewe lambs, especially in seasonal anestrus females (Salehi et al., 2010). Some differences have been known in ewe lambs compared to adult ewes in oestrus, ovulation and the length of breeding season. It has been reported that ewe lambs in her first breeding season has a lower ovulation rate compared to adult ewes (Scaramuzzi & Radford, 1983). However, the lower body weight of the ewe lambs together with other differences in their nutrition further confounds this relationship. Furthermore, ewe lambs show oestrus for a shorter time than adult ewes (Signoret & Cognie, 1975). In addition, the breeding season of ewe lambs typically begins 3 weeks after the adult ewes and ceases 3 weeks prior to the adult ewes (Youngquist & Threlfall, 2007). However, the information about ovulation distribution following ES in ewe lambs, especially during non‐breeding season, is nadir. Therefore, decision‐making about the effect of a treatment on intensive synchrony of ovulation in varying herds with different managements seems complicated. The limited studies have been conducted on the determination of ovulation after the removal of intravaginal progesterone devices. We hypothesized that the ovulation distributions would be different following synchronization with different vaginal devices, and it could affect the results of pregnancy outcomes. The present study aimed to assess the distribution of OVT (every 3–4 h, with transvaginal ultrasonography) following synchronization with CIDR or MPA vaginal sponge devices + eCG, and its effect on the conception following FTAI with frozen/thawed semen samples in fat‐tailed Qezel ewes outside the breeding season.

## 2 MATERIALS AND METHODS

### 2.1 Study region and animal care management

The current study was conducted on Qezel ewes (*n* = 12; 34.68 ± 1.11 months of age; 64.14 ± 2.88 kg body weight) and ewe lambs (*n* = 10; 11.16 ± 0.64 months of age; 51.41 ± 1.67 kg body weight) outside the breeding season (May–June). The ewes were kept under uniform management condition at the small ruminant farm belonging to the Agriculture Faculty of Urmia University, West Azerbaijan, Iran. The animals were flushed with alfalfa and concentrate (soybean, maize, oat, enzymite, carbonate, salt and molasses) for 2 weeks before synchronization. During the flushing period, all animals received AD_3_E vitamins (5 mL, Eurovet Animal Health BV) and E.sel combination (5 mL, Eurovet Animal Health BV) as a subcutaneous injection.

### 2.2 Synchronization protocol

**FIGURE 1:**
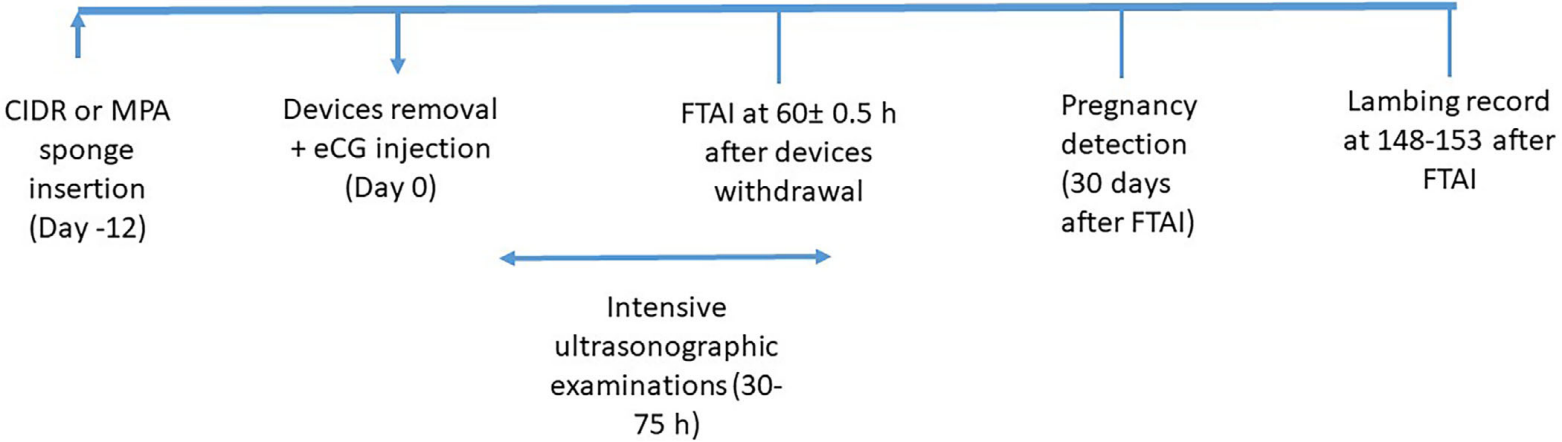
The time line of experiment.

The ewes and ewe lambs were randomly divided into two groups according to the synchronization method. Ewes (*n* = 6) and ewe lambs (*n* = 5) of the first group received an intravaginal sponge containing 60 mg MPA (Sponjavet, Hipra; Day −12 of the study), and the animals in the second group (*n* = 11; six ewes and five ewe lambs) treated with CIDR devices (Eazi‐breed CIDR, Zoetis; containing 0.3 g progesterone). Both intravaginal devices remained for 12 days, and at removal, all females received an intramuscular dose of 500 IU eCG (Gonaser, Hipra; Day 0 of experiment). Time line of experiment is depicted in Figure 1.

### 2.3 Ultrasonographic evaluation of ovaries via a transvaginal approach

The ovaries of all females were examined using real‐time, B‐mode ultrasonography equipped with a 9 MHz transvaginal transducer (Emperor, EMP 830Vet) at Days −12 (device insertion) and 0 (device removal + eCG administration; DesCôteaux et al., 2009). Furthermore, intensive ovarian examination was restarted at 30 h and continued every 3–4 h until 75 h after device removal + eCG administration in all females. During the ultrasonographic examination, the ewes were haltered in a box within a dark room while standing. Before insertion of the probe, the external genitalia was completely cleaned and disinfected with an alcohol (70%) pad. The presence, number and diameter (horizontal and vertical) of the ovarian follicles greater than 1 mm were recorded on individual case report forms for each animal and tabulated for growth. The ovulation was confirmed by the disappearance of a large, growing follicle that was tracked several times, followed by the formation of the corpus luteum in the referred ovary. The presence, number, location (left or right ovary) and diameters (horizontal and vertical) of the corpora lutea were assessed 11 days after device removal + eCG injection (D +11).

### 2.4 Blood collection and progesterone analysis

Blood samples were collected from the jugular vein in vacuum tubes (VACUETTE, Bio‐one GmbH) on Days of −12, 0, +2 (day of insemination) and +11 of the experiment in all females. Sera samples were separated through centrifugation (Hettich) at 3000*g* for 30 min. The sera samples were collected in 2 mL microtubes and stored at −20°C until progesterone assay. The progesterone (P4) levels were measured using an ELISA kit (Sheep progesterone ELISA kit, ZellBio; sensitivity = 0.06 ng/mL; intra‐assay CV < 4.4%; and inter‐assay CV < 7.1%) according to the instructions of manufacture. Then the optical density of the contents was read at 450 nm against blank sample using an ELISA reader.

### 2.5 Semen samples collection and freezing

Semen samples of the fertile ram (two ejaculations) were collected using an artificial vagina (IMV) in the presence of an oestrus ewe. The samples with spermatozoa mass motility greater than 3, progressive motility >70% and spermatozoa count greater than 3 × 10^9^/mL were selected for cooling/freezing. The kinematic values of spermatozoa were analysed using the CASA system (Videotest; Test Sperm 3.2). Spermatozoa motility was recorded at 50 frames/s by the software. To evaluate the kinematics, a 10 μL sample was placed on a preheated slide (37°C), covered with a cover slide (22 × 22 mm) and examined under a phase contrast microscope (Labomed Inc.). Immediately after collection, the samples were diluted (at 400 × 10^6^ motile spermatozoa/mL) with an extender containing tris, citric acid, fructose, glycerol, penicillin‐streptomycin and plasma egg yolk (28%) (Mortazavi et al., 2020) and then were filled in 0.25 mL straws (Minitube). The final concentrations of tris, citric acid, fructose and glycerol were adjusted to 299.65, 103.60 and 27.77 mM as well as 5%, respectively (Salamon and Maxwell, 2000), in the diluent. The straws (contains 100 × 010^6^ motile spermatozoa) were sealed with polyvinyl alcohol, slowly cooled from 37 to 4°C in 3.5–4 h and kept in a 4°C refrigerator for additional 2 h. They were then placed horizontally in a styrofoam container at 4 cm above liquid nitrogen for 15 min subsequently immersed in liquid nitrogen. In order to evaluate the quality of frozen samples, two straws were thawed in a 37°C water bath for 2 min, and then the contents were analysed using the CASA system. The mean motilities of spermatozoa after freezing–thawing were 67.21%.

To extraction of plasma egg yolk (according to MacBee and Cotterill, 1979, with some modification), an equal volume of egg yolk was mixed with tris–citric acid–fructose diluent and stirred for 1 h at 4°C. The solution was centrifuged (Hettich) two times at 9000*g* at 2–5°C for 45 min. Following the second centrifugation, the upper layer (egg yolk plasma) was carefully collected using a pipette, stored at 4°C and used for extender preparation.

### 2.6 Artificial insemination

Intrauterine insemination was carried out in all females, 60.5 ± 0.5 h after withdrawal of the intravaginal progestagen devices through laparoscopy (Maxwell, 1986; Walker et al., 1989). The females were placed in the supine position on a laparoscopic bed with the ability of 90° rotation. To introduce the light source‐attached laparoscope (Stroz), and the insemination pipette, two trocars (5 mm; Stroz) were placed on both sides of the abdominal midline, 2–3 cm apart from the midline. A regulator was directly connected to the CO2 tank to rapidly fill the abdominal cavity before insemination. Then the two straws were thawed, mixed and inseminated in the middle part of the greater curvature of each uterine horn. Each uterine horn received more than 50 × 10^6^ motile frozen‐thawed spermatozoa.

### 2.7 Pregnancy evaluation

The presence and number of live embryos were recorded by ultrasonographic examination via transvaginal approach, 30 days after FTAI. The conception rate was characterized by the number of pregnant ewes on day 30/total number of inseminated ewes × 100.

### 2.8 Statistical analysis

Effect of treatment and time on progesterone concentrations were analysed using two‐way ANOVA. The mean time to ovulations (effect of vaginal devices × parity or vaginal devices × number of ovulations) and mean sizes of ovulatory follicles and corpora lutea were analysed using two‐way ANOVA. The Holm‐Sidak procedure was used as a post hoc test. Pregnancy outcomes were assessed using chi‐square test. The SigmaStat software (Version 3.5) was used to analyse the data. A *p*‐value less than 0.05 was considered to be significant, and a *p*‐value between 0.05 and 0.10 was used to express any tendency of data towards significance.

## 3 RESULTS

**FIGURE 2:**
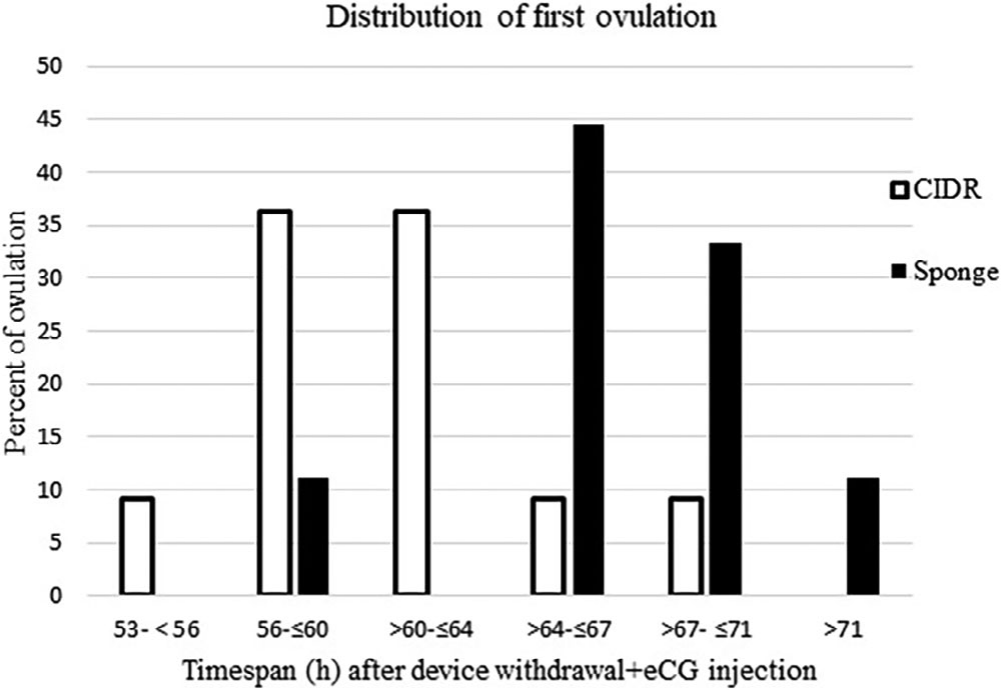
Distributions of first ovulation in fat‐tailed Qezel females at different timespans after oestrus synchronization with controlled internal drug release (CIDR)/vaginal sponge + equine chorionic gonadotropin (eCG), outside the breeding season. ‘□’ shows the CIDR, and ‘■’ represents the sponge received group, respectively.

**FIGURE 3:**
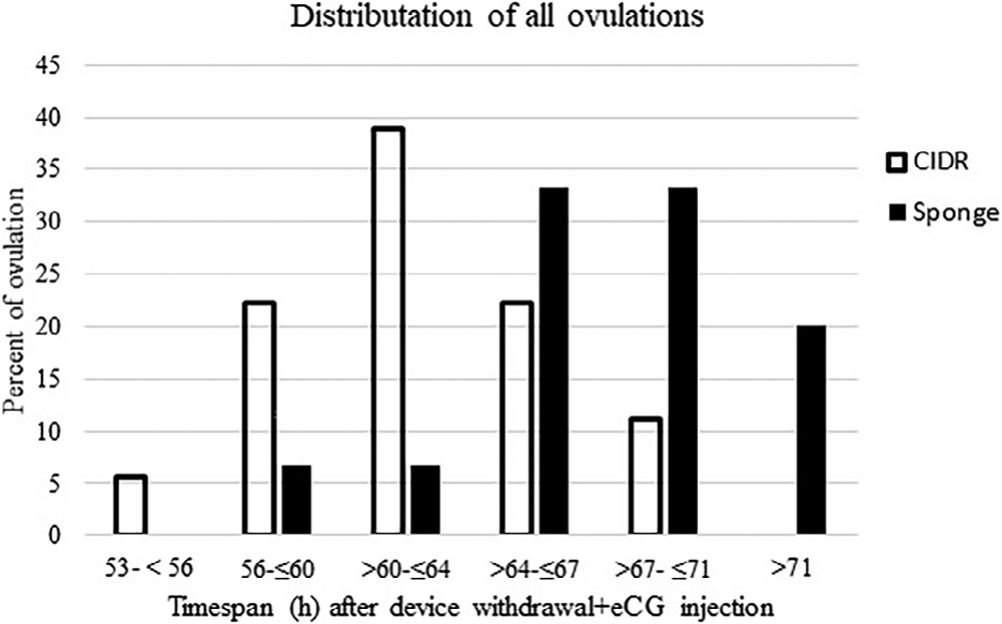
Distributions of all ovulation in fat‐tailed Qezel females at different timespans after oestrus synchronization with controlled internal drug release (CIDR)/vaginal sponge + equine chorionic gonadotropin (eCG), outside the breeding season. A total of 18 and 15 follicles were ovulated from 11 and 10 females in the CIDR and sponge groups, respectively. ‘□’ shows the CIDR, and ‘■’ represent the sponge received group, respectively.

One ewe belonging to the vaginal sponge group was excluded from the study due to showing signs of fever and inappetence on the Day +1. Of the 21 ewes and ewe lambs that remained in the study, 20 animals ovulated, and 1 ewe lamb did not ovulate following ES with vaginal sponge + eCG treatment and did not have any corpus luteum on ovaries at Day 11. The ovulations initiated and terminated earlier in the CIDR‐treated animals compared to sponge‐treated animals, that is (53–56 vs. 56–60 h) and (67–71 vs. 71–75 h), respectively (Figures 2 and 3).

In CIDR‐treated group, the median time of first (64 vs. 69 h) and all (64 vs. 71 h) ovulations and mean time of first (62.72 vs. 69.10 h) and all (64.11 vs. 69.26 h) ovulations were less (*p* < 0.05) than sponge‐treated females (Table 1, Figures 2 and 3).

**TABLE 1:** Ovulatory performance following oestrus synchronization using controlled internal drug release (CIDR)/vaginal sponge + equine chorionic gonadotropin (eCG) in fat‐tailed Qezel ewes outside the breeding season.

Groups (number of ewes)	Number of ovulatory follicles	Mean sizes of ovulatory follicles (mm)	Median time of first ovulation (h)	Mean (±SEM) time of first ovulation (h)	Median time of all ovulation	Mean (±SEM) time of all ovulation (h)	Mean interval from first to last ovulations (min‐max)
CIDR (*n* = 11)	18	7.2 ± 0.22	64	62.72 ± 1.03	64 h (18)	64.11 ± 0.80	2.8 ± 1.90 (0–11)
Sponge (*n* = 10)	15	7.58 ± 0.23	69	69.10 ± 1.34	71 h (15)	69.26 ± 1.06	2.01 ± 1.24 (0–8)
*p* Value	0.93	0.24	0.006	0.006	<0.001	<0.001	0.93

*Note*: Vaginal sponge contains 60 mg medroxyprogesterone acetate.

Abbreviations: CIDR, controlled internal drug releasing devices; eCG, equine chorionic gonadotropin.

The mean and median OVTs did not differ between ewe (*n* = 6) and ewe lambs (*n* = 5) in CIDR‐treated group. Ewe lambs (*n* = 4) ovulated earlier compared to ewes (*n* = 5) in sponge‐treated group (66.71 vs. 71.5; *p* = 0.023; Table 2). The mean OVT was recorded later in sponge‐treated ewes (71.5 ± 1.10 h) compared to CIDR‐treated ewes (62.55 ± 1.16 h; *p* < 0.001) and ewe lamb (63.66 ± 0.85 h; *p* < 0.001). Furthermore, sponge‐treated ewe lambs ovulated later (66.71 ± 1.35 h) compared to CIDR‐treated ewes (*p* = 0.046). The comparison between two ewe lambs groups indicated that CIDR‐treated ewe lambs tend to be ovulated earlier (63.66 ± 0.85 h) compared to sponge‐treated ewe lambs (*p* = 0.10).

**TABLE 2:** Ovulatory performance of fat‐tailed Qezel females (ewe vs. lamb; mono‐ vs. multi‐ovulators) following oestrus synchronization using controlled internal drug release (CIDR)/vaginal sponge + equine chorionic gonadotropin (eCG) outside the breeding season.

Groups (number of ewes)		Median, mean (±SEM) time of ovulation (h)	*p* Value	Mean sizes of ovulatory follicle (mm)	*p* Value
CIDR (*n* = 11)	Ewe vs. lamb	Ewe = 60, 62.55 ± 1.16 Ewe lamb = 64, 63.66 ± 0.85	0.52	Ewe = 7.30 ± 0.31 Ewe lamb = 7.11 ± 0.31	0.67
CIDR (*n* = 11)	Mono vs. multi‐ovulators	Mono = 60, 61.83 ± 1.66 Multi = 64, 65.25 ± 1.06	0.08	Mono = 7.61 ± 0.40 Multi = 6.99 ± 0.26	0.42
Sponge (*n* = 10)	Ewe vs. lamb	Ewe = 71, 71.5 ± 1.10 Ewe lamb = 67, 66.71 ± 1.35	0.017	Ewe = 7.85 ± 0.35 Ewe lamb = 7.28 ± 0.20	0.23
Sponge (*n* = 10)	Mono vs. multi‐ovulators	Mono = 67, 68.33 ± 1.08 Multi = 71, 69.50 ± 1.28	0.68	Mono = 7.96 ± 0.35 Multi = 7.49 ± 0.25	0.44

Abbreviations: CIDR, controlled internal drug releasing devices; eCG, equine chorionic gonadotropin. Vaginal sponge contains 60 mg medroxyprogesterone acetate.

There was no significant difference between mono‐ and multi‐ovulatory sponge‐treated females. On the other hand, females of the CIDR‐treated group with single ovulation tended to ovulate earlier (after device removal) than the multi‐ovulatory females (61.83 vs. 65.25; *p* = 0.08; Table 2).

Mean sizes of ovulatory follicles did not differ between CIDR and sponge‐treated groups, or between ewe and ewe lambs, and between mono and multi‐ovulatory females of within each group (Tables 1 and 2).

The mean size of corpora lutea was not affected by the type of progesterone releasing device (CIDR: 12.41 mm; vaginal sponge: 12.47 mm), and between ewe and ewe lambs or mono vs. multi‐ovulatory females of each group (*p* > 0.05).

**FIGURE 4:**
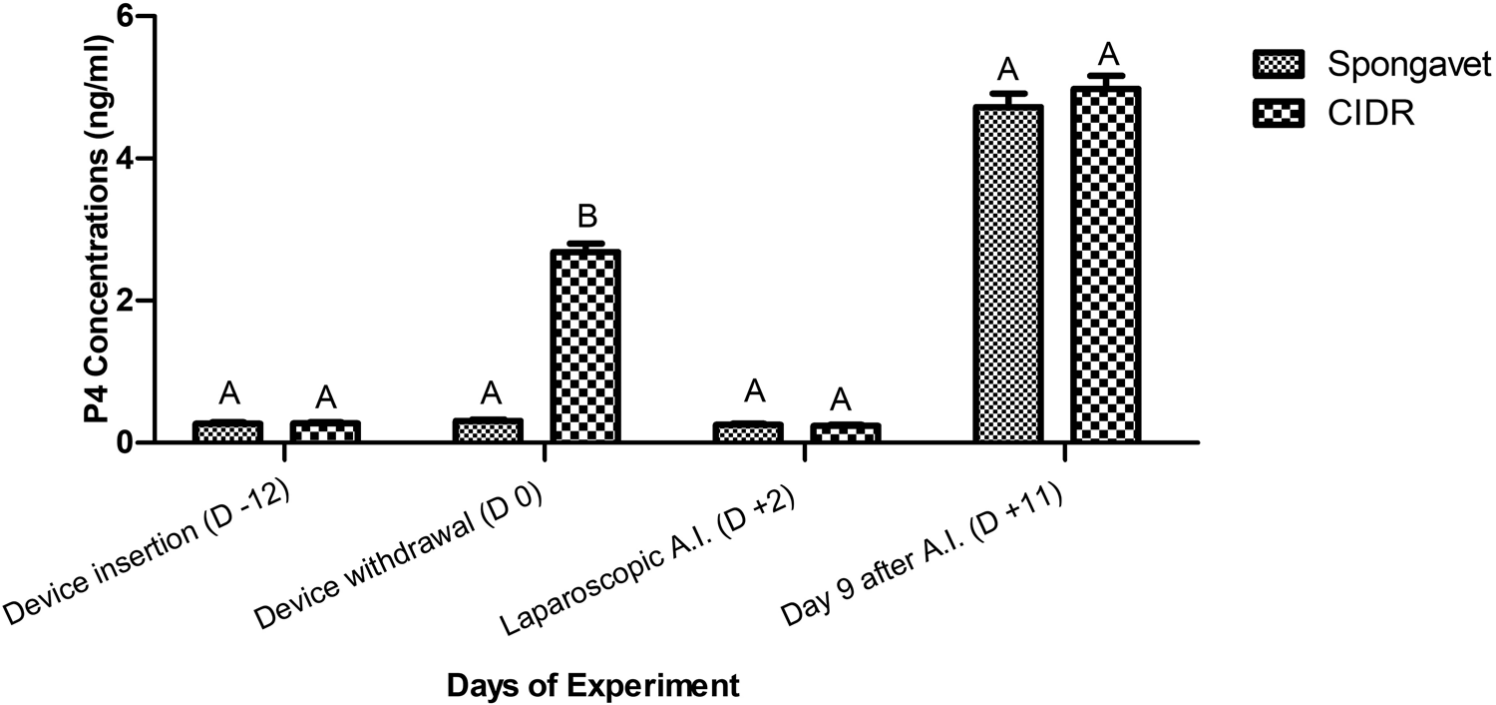
Mean progesterone (P4; ng/mL) concentrations of controlled internal drug release (CIDR) and sponge received groups at device insertion (−12), device withdrawal (0), laparoscopic artificial insemination (A.I.) (+2), and Day 9 after insemination (D +11 of experiment). Higher progesterone amounts were detected in CIDR received group at the time of device removal compared to sponge primed females. ‘□’ shows the CIDR, and ‘■’ represents the sponge received group, respectively.

Mean progesterone concentrations of sponge‐treated group were 0.26 ± 0.02, 0.30 ± 0.01, 0.25 ± 0.01 and 4.72 ± 0.19 ng/mL at the time of device insertion, device removal, laparoscopic insemination and Day 11, respectively (Figure 4). Furthermore, CIDR‐treated animals showed amounts of progesterone similar to sponge‐treated females at the time of device insertion (0.27 ± 0.01 ng/mL), laparoscopic A.I. (0.23 ± 0.01 ng/mL) and Day 11 (4.97 ± 0.2 ng/mL; *p* > 0.05). Higher amounts of progesterone were detected in CIDR‐treated group compared to sponge‐treated females at the time of device withdrawal (*p* < 0.001; Figure 4).

Following laparoscopic insemination with frozen‐thawed semen sample (at 60.5 ± 0.5 after device removal), the conception was confirmed in 2 (2/10 = 20%) and 5 (5/11 = 45.45%) females of sponge and CIDR‐treated groups, respectively (Table 3). The conception was closely related to the synchrony of ovulation and insemination during every 3–4 h assessment. According to the CIDR‐treated outcomes, the best time for insemination is a few hours before ovulation.

**TABLE 3:** Number of ovulations in different timespan and conception following laparoscopic insemination at 60.5 ± 0.5 h after device removal + equine chorionic gonadotropin (eCG) administration.

Time of ovulation	Type of vaginal device	Number of ewes ovulated	Number of ovulated follicles	Number of pregnant ewes (%)	Number of lams born
53–<56	CIDR	1	1	0 (0)	0
	Sponge	0	0	0	0
56–≤60	CIDR	4	4	1 (25)	1
	Sponge	1	1	1 (100)	2
>60–≤64	CIDR	4	7	3 (75)	4
	Sponge	0	1	0	0
>64–≤67	CIDR	1	4	1 (100)	1
	Sponge	4	5	1 (25)	1
>67–≤71	CIDR	1	2	0 (0)	0
	Sponge	3	5	0	0
>71–75	CIDR	0	0	0 (0)	0
	Sponge	1	3	0	0

*Note*: Total conception rate: CIDR = 45.45% (5/11); vaginal sponge = 20% (2/10).

Abbreviations: CIDR, controlled internal drug releasing devices; eCG, equine chorionic gonadotropin. Vaginal sponge contains 60 mg medroxyprogesterone acetate.

## 4 DISCUSSION

Current experiment was conducted to reveal the distribution of ovulation in the non‐cyclic fat‐tailed Qezel ewe and ewe lambs by serial intensive transvaginal ultrasonographic examinations following synchronization with vaginal loaded progestagen devices (CIDR vs. MAP sponge) + eCG. Furthermore, the relation of conception with OVT was assessed after intrauterine insemination with frozen‐thawed semen samples.

The FTAI has numerous advantages in the management of ewe breeding; however, one of the main problems associated with A.I. is the accurate estimation of OVT, which is necessary for performing in‐time insemination, and maximizing fertility (Romano, 1996). In this regard, Maxwell. (1986) indicated that the median time of ovulation in the mature cyclic Merino ewes following synchronization with 30 mg flugestone acetate (FGA) in combination with eCG therapy at sponge removal in unteased ewes was 59.7 h (58.27–61.12) after hormone injection. Furthermore, the mean ovulation rates were 1.8 and 1.6 for teased and untease ewes, respectively. Results of the present study displayed that the median and mean times of first and all ovulations were lower (in relation to eCG injection) in CIDR‐treated ewes compared to sponge‐treated cases. Walker et al. (1989) revealed that CIDR‐treated Merino ewes ovulated earlier (median time = 51 h) compared to MPA‐treated ewes (median time = 69 h), which is compatible with our results. Previous studies evaluated the ovulation and the number of ovulated follicles, using laparoscopic lens examination (Maxwell, 1986; Walker et al., 1989). The efficacy of laparoscopic lens for the evaluation of the exact time of ovulation was under question. Current experiment assessed the ovulation by serial intensive ultrasonographic examination via transvaginal approach with high resolution (9 MHz) frames. It seems that the method of ovulation assessment would affect the results.

The CIDR is formed of a nylon core and surrounded by natural progesterone‐containing silicone elastomer containing 300 mg of endogenic progesterone and the sponge, which contain exogenous progestagen made of FGA or 60 mg MPA (Menchaca et al., 2007). Not only the characteristics of progestagens differ between MPA and CIDR, but also the differences in physical forms (sponge vs. CIDR) resulted in the variation in the absorbances of progesterone, especially during device withdrawal (Ryan & Rosner, 2001). The physical pressure induces during sponge withdrawal exudes the progestin from the sponge and extending the exposure of hormone within the vagina and its effects following absorbances. While, by removing the CIDR, the amounts of progesterone decrease abruptly (Bartlewski et al., 2015). It was indicated that clearance rate of the synthetic exogenous progestagens such as MPA take more time compared to endogenous analogues of natural progesterone (Husein & Kridli, 2002). The mentioned reasons (physical differences and more half‐life) justify the delayed ovulations in sponge‐treated ewes compared to CIDR received group (71 vs. 64 h after removal). In the current study, progesterone concentrations at device withdrawal were significantly higher in CIDR‐treated group (2.68 ± 0.12 ng/mL) compared to MPA sponge‐treated group (0.30 ± 0.01 ng/mL). It seems that progesterone assay kit used in the present study could not cross react with the exogenous progestagens (MPA) released from the vaginal sponge. This finding is compatible with the previous report (Husein & Kridli, 2002).

According to the relationship of OVT (estimated by laparoscopic lens) and conception rate, Maxwell (1986) concluded that laparoscopic insemination was more effective when performing after ovulation. They reported that the median range of ovulations were 54.61–57.09 and 58.27–61.12 h for teased and unteased cyclic ewes after FGA sponge withdrawal, respectively. However, laparoscopic insemination with frozen‐thawed samples at 48, 60, 72 and 78 h after sponge withdrawal resulted in 46%, 55%, 57% and 39% pregnancies in ewes (Maxwell, 1986). Consequently, the author revealed that even 16–22 h after ovulation, the fertilizing ability of sheep ovum is acceptable. A previous study reported that the fertilizing ability of sheep ovum was 12–24 h after ovulation (Dzuik, 1965). Controversial findings were reported about the relation of ovulation distributions, insemination time and the conception rate. Fertility was reported higher in ewes inseminated 44 h (63%; before initiation of ovulation) relative to 68 h (38%; after ovulation) following FGA withdrawal (Maxwell et al., 1993). However, it was reported significantly higher when frozen‐thawed semen was deposited in the uterus at 60 h (60%) compared to 52 h (20%) after progestagen removal (Martemucci & D'Alessandro, 2011). In other words, insemination 1‐h before the estimated OVT (53 h after progestagen removal) decreased the conception rate, which is similar to our results and others (Maxwell, 1986). The proposed reasons of lower fertility following performing laparoscopic insemination around the time of ovulation were probably due to interference with the normal rupturing of pre‐ovulatory follicles and successful collection of the oocytes (Robinson et al., 1989), or impairment of ova transition toward the fertilization site of the oviduct (Maxwell et al., 1993). Furthermore, the synchronization of non‐cyclic ewes with CIDRs or MPA sponges, and consequently intrauterine insemination 60 h after progestagen withdrawal, resulted in 40% and 46% conception rate, respectively (Fukui et al., 1993). However, authors primed animals 9‐day with progestagen, and the eCG was administrated a day before device withdrawal which could affect the follicular growth and the time of ovulation compared to the injection of the hormone at progestagen removal. However, insemination at 48 h after progestagen removal caused higher conception rate in CIDRs‐treated groups (73%) compared to MPA sponges primed ewes (52%; Fukui et al., 1993). Literature revealed that 8 h (Martemucci & D'Alessandro, 2011) or 16 h (Maxwell, 1986) after ovulation, the conception rate following laparoscopic insemination would be acceptable (55%–60%), which was not observed in our experiment, especially in CIDR‐treated females. The recommended timespan for laparoscopic insemination following synchronization is 48–72 h after vaginal sponge removal + eCG administration. Insemination before (24–36 h) or after (78 h) the mentioned period reduced the conception rate (Maxwell et at, 1984; Maxwell, 1986; Jabbour & Evans, 1991). The mentioned results verify the earlier ovulation in CIDR‐treated ewes compared to MPA primed females, as seen in our experiment and other (Walker et al., 1989). Furthermore, current study displayed that early or late insemination of frozen‐thawed semen sample relative to OVT (±7 h) reduced the conception rate, due to the ageing of spermatozoa or ova, respectively, in the female genital tract (Maxwell et al, 1984). However, a total number of 22 females were allocated in the CIDR and sponge groups, and a higher number of females are needed to confirm the results with greater power of test.

Current study confirms the earlier ovulation following synchronization with endogenous progesterone releasing devices compared to exogenous synthetic progestagen releasing sponges in ewe and ewe lambs during non‐breeding season. The optimum timespan for laparoscopic insemination with frozen‐thawed semen samples should be chose according to the types of utilized vaginal devices. Current study performed the FTAI 60.5 ± 0.5 h after CIDR or vaginal sponge removal in a small number of females, further studies with greater number of females are required to confirm the current findings.

## AUTHOR CONTRIBUTIONS

Alireza Hamisi collected and analysed the semen samples, assisted in the ultrasonographic examination and laparoscopic insemination and measured the progesterone amounts by ELISA kit; Mohsen Eslami designed the experiment, performed the laparoscopic insemination, involved in the evaluation and analysis the data and wrote the manuscript; Farhad Farrokhi‐Ardabili involved in the oestrus synchronization, freeze the semen samples and supervised the study; Sina Bahmani involved in the sampling and synchronization, and edited the manuscript. All authors have read and agreed to be listed in the manuscript.

## CONFLICT OF INTEREST STATEMENT

The authors had no conflicts of interest.

## FUNDING INFORMATION

The current study was supported by the Research Deputy of Urmia University (Grant/Award Number: ‘TD/6915’) and the Dam Zist Kara Sabz (Grant/Award Number: ‘1401/09/2’) company.

### PEER REVIEW

The peer review history for this article is available at https://www.webofscience.com/api/gateway/wos/peer‐review/10.1002/vms3.1269.

### ETHICS STATEMENT

Semen sampling process, transvaginal examination and laparoscopic insemination were verified by the care committee of Urmia University (IR‐UU‐AEC‐3/PD/1745).

## Data Availability

Data available on request due to privacy/ethical restrictions.
